# Ultrastructure Characteristics and Sexual Dimorphism of Antennal Sensilla in *Tirathaba rufivena* (Lepidoptera: Pyralidae)

**DOI:** 10.3390/insects13090797

**Published:** 2022-09-01

**Authors:** Jixing Guo, Zimeng Du, Guangchao Cui, Zheng Wang, Junfang Wang, Xiang Zhou

**Affiliations:** Key Laboratory of Green Prevention and Control of Tropical Plant Diseases and Pests, Ministry of Education and College of Plant Protection, Hainan University, Haikou 570228, China

**Keywords:** *Tirathaba rufivena*, sensilla structure, sexual dimorphism, scanning electron microscopy, transmission electron microscopy

## Abstract

**Simple Summary:**

To elucidate the external morphology and internal structures of antennal sensilla that could play a role in locating host plants and mates, we describe and further characterize the ultrastructure, abundance and distribution of nine sensilla types in both sexes using scanning electron microscopy and transmission electron microscopy. Sexual dimorphism mainly occurs in variation in the length of sensilla basiconica, sensilla chaetica, sensilla coeloconica 1 and Böhm sensilla, and the abundance of sensilla basiconica, sensilla auricillica 1 and sensilla auricillica 2. The possible structural function of each kind of sensillum is discussed. These results provide direct morphological evidence that the antennae of *Tirathaba rufivena* possess structures that can play a role in locating mates and host plants.

**Abstract:**

*Tirathaba rufivena* Walker, a major insect pest of *Areca catechu* L., has severely threatened areca nut cultivation in Hainan, China. To improve our understanding of the communication mechanism in host plant seeking and mate-finding for *T. rufivena*, we described and further characterized the external morphology and internal sensilla structures using scanning electron microscopy and transmission electron microscopy in this study. The antennal morphology was similar between males and females, and there was no significant difference in length between the two sexes. In total, nine sensilla types were identified: sensilla trichodea (Str), sensilla chaetica (Sch), sensilla basiconica (Sba), sensilla auricillica (Sau), sensilla coeloconica (Sco), sensilla styloconica (Sst), Böhm sensilla (Bs), uniporous peg sensilla (Ups) and sensilla squamiformia (Ssq). Sexual dimorphism mainly occurs in variation in the length of Sba, Sch, Sco1 and Bs, and the abundance of Sba, Sau1 and Sau2. The Sba had larger size and numbers on female antennae than that on males, suggesting that these sensilla might have important roles in locating host plants. Both Sau1 and Sau2 were significantly more abundant in females and were probably associated with the detection of mates and host plant for oviposition. These data were important for ongoing studies on host plant seeking and mate-finding behavior in *T. rufivena* and provided a theoretical foundation to further studies of semiochemical control for this pest.

## 1. Introduction

Insect olfaction plays an important role in the process of host seeking, mate finding and enemy avoidance. The antennae, which are covered with numerous sensilla, are the primary sense organs in insects. Olfactory sensilla are used to detect odor molecules in the environment, such as plant volatiles and pheromones [1,2,3,4,5,6]. Sensilla have different kinds of morphologies and diverse functions. Based on their external appearance and structure, sensilla can be classified into different types, including trichodea, chaetica, auricillica, basiconica, coeloconica, squamiformia, styloconica, placodea, ampullacea, campaniform, uniporous peg and Böhm sensilla [7,8]. By now, the ultrastructure of antennal sensilla in various insect species has been described and characterized to reveal the mechanism of olfaction using scanning electron microscopy (SEM) and transmission electron microscopy (TEM) e.g., [9,10,11,12,13,14,15,16].

Different insect species possess diverse sensilla types, in different relative abundances. The lepidopteran insects may have tens of thousands of sensilla, which are classified into several subsets of morphological types [8,9,17]. The different sensilla types are used for specific functions. The sensilla trichodea, basiconica, auricillica, coeloconica and placodea are used for olfaction, sensilla chaetica for taste (uniporous) or touch (without pores). The sensilla ampullacea and styloconica are mainly involved in thermo or hygroreception, and Böhm sensilla, sensilla campaniform and squamiformia in mechanoreception [8]. 

Sexual dimorphism of antennal sensilla has been reported in many studies. For some insects, males have a larger type, number, or region than females. Such observations could indicate that these sensilla may play a role in sexual communication [5,18]. Males are the recipients of attractive volatile molecules because females generally release pheromones [19,20,21,22]. In other species, however, females have longer antennae, comprised of a higher number of shorter segments than males in *Galleria mellonella*. Female antennae processed a higher abundance of sensilla than male antennae, which supports specific sexual behavior of females of *G. mellonella* [23]. Moreover, similar findings have been reported in *Plodia interpunctella* and *Lobesia botrana*. The antenna has greater flagellar length and abundance in males compared with that of females [24,25].

*Tirathaba rufivena* Walker (Lepidoptera: Pyralidae), commonly known as the coconut spike moth, is a major insect pest of Arecaceae species, such as *Areca catechu* L., *Cocos nucifera* L., and *Elaeis guineensis* Jacq [26,27]. The damage caused by *T. rufivena* has severely threatened areca nut cultivation in Hainan, China, with high damage rates [28,29]. The major effective method to control this pest still relies on chemical pesticides, which not only results in environmental pollution but also has bad effects on human health [30]. Volatiles from areca inflorescence plays a communication and guide role in host localization for *T. rufivena* [31]. Olfactory sensilla play important roles in this process. Huang et al. played the SEM on the egg and antenna of *T. rufivena* [32]. A total of seven types of sensilla were identified and analyzed, while the internal structure and function were still not clear. To improve our understanding of the communication mechanism, we described and further characterized the external morphology and internal sensilla structures of *T. rufivena* using SEM and TEM. The ultrastructural characteristics, number and distribution of different sensillum types in both sexes were studied to document differences between females and males. In addition, the possible structural function of each kind of sensillum was also discussed. These data are important for ongoing studies on host plant seeking and mate-finding behavior in *T. rufivena* and provides a theoretical foundation to further studies of semiochemical control for this pest.

## 2. Materials and Methods

### 2.1. Insect Rearing and Collection

The *T. rufivena* larvae were collected from an areca field and kept at 25 ± 1 °C and 70 ± 5% relative humidity with a light: dark cycle of 16:8 h at the College of Plant Protection, Hainan University, Haikou, China. The larvae were reared on an artificial diet. Pupae were collected, sexed and then separated. For the present experiments, 2–3 day old females and males were used for examination.

### 2.2. Scanning Electron Microscopy

Ten adult males and ten adult females (2–3 days old) of *T. rufivena* were narcotized at −20 °C. The heads of the male and female were dissected from the body and longitudinally cut in half by fine scissors and immersed in glutaraldehyde (2.5%) at 4 °C for SEM. The samples were first washed with PBS (0.1 M, pH = 7.4) 3 times and postfixed in 1% osmium tetroxide in PBS at 4 °C for 2 h. A graded series of ethanol (30%, 50%, 70%, 80%, 95%, and 100%) was used to dehydrate the antenna. Then, the critical point dried antennal samples were mounted on stubs with double-sided adhesive tape, and sputter-coated with gold: palladium (3:2) and visualized using SU8100 SEM (Hitachi, Tokyo, Japan).

### 2.3. Transmission Electron Microscopy

The antennae were fixed for 24 h in 2.5% glutaraldehyde with 0.1 M sodium cacodylate at pH 7.4. Then, the samples were rinsed in 0.1 M cacodylate buffer and then post-fixed in 1% osmium tetraoxide in 0.1 M cacodylate buffer for 2 h, all at pH 7.0 and 25 °C. The samples were then rinsed four times in distilled water (4 × 15 min) and dehydrated for 15 min each, in a graded ethanol series of 30%, 50%, 70%, 90%, and changed twice for 20 min each, in 100% ethanol. A oven (60 °C, 48 h) was used to polymerize the antennal samples after they were embedded in epoxy resin. Serial sections of 50–70 nm were collected on formvar-coated nickel grids after the specimen was cut with a diamond knife on a Leica CM1950 platform (Leica, Nussloch, Germany). Sections were stained with uranyl acetate and lead citrate according to the conventional method. The sample grids were observed using a Hitachi HT7700 TEM (Hitachi, Tokyo, Japan).

### 2.4. Data Processing and Statistical Analysis

The classification and types of sensilla identified in this study were based on Schneider and Callahan [33,34]. The antennal length and the number, length, diameter and distribution of each type of sensilla were analyzed from SEM and TEM micrographs. The lengths of the antennal segments and sensilla were measured with Image-Pro Plus software (Media Cybernetics, Rockville, MD, USA). The total number of sensilla trichodea was calculated according to the method reported by Roh [35]. The length of at least 15 similar sensillum types were measured and analysed to determine the presence of sexual dimorphism. Means were calculated and statistically analyzed by a Student’s *t*-test using SPSS Statistics 18.0 (IBM, Armonk, NY, USA) to determine significant signs of sexual dimorphism. The images were enhanced and composed using Adobe Photoshop CS6 (Adobe, San Jose, CA, USA).

## 3. Results

### 3.1. Antennal Morphology

**Figure 1:**
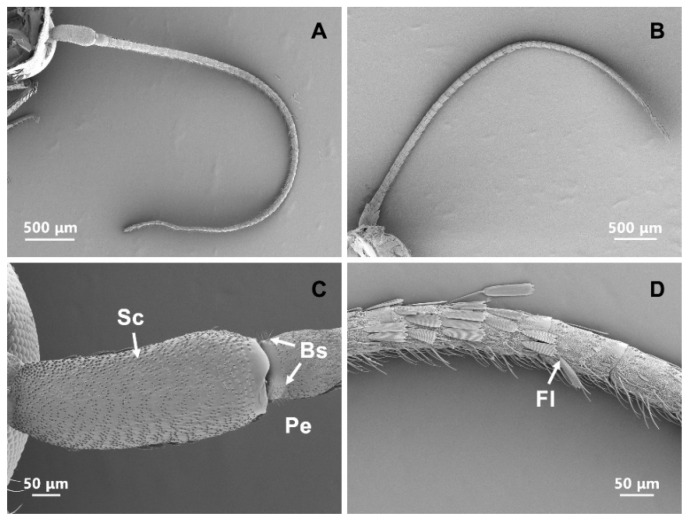
Scanning electron micrographs of the entire antenna of *T. rufivena*. (**A**) Overview of the antenna of female *T. rufivena*; (**B**) overview of the antenna of male *T. rufivena*; (**C**) the scape and pedicel segments of the antenna with scales removed; (**D**) flagellar segments of the antenna with scales partly removed. Sc: Scape; Pe: pedicel; Fl: flagellum.

The gross antennal morphology was similar between male and female *T. rufivena* (Figure 1A,B). The antennae were filiform and composed of three segments: a proximal scape, a pedicel and a distal flagellum with 40–45 subsegments (Figure 1C,D).

**Figure 2:**
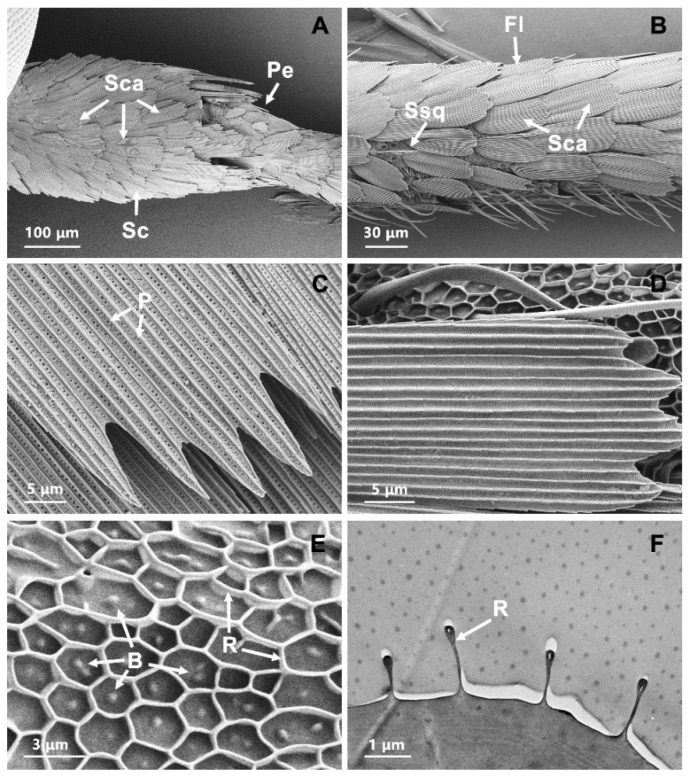
Scanning electron micrographs and transmission electron micrographs of scales and the reticular surface. (**A**) Distribution characters of scales on scape and pedicel segments; (**B**) distribution characters of scales on flagellar segments; (**C**) the structure characters of scales on scape and pedicel segments; (**D**) the structure characters of scales on flagellar segments; (**E**) the reticular network structure on the flagellar segment; (**F**) longitudinal section of ridges extended over the antennal surface. Sc: scape; Pe: pedicel; Fl: flagellum; Sca: scale; P: pore; R: ridge.

The scape was 499.27 ± 26.96 μm in length and 208.19 ± 26.02 μm in diameter at the base. The pedicel was 139.74 ± 16.49 μm in length and 115.49 ± 6.36 μm in diameter at the base. There was no significant difference in antennal length between females (5.39 ± 0.20 mm) and males (5.23 ± 0.19 mm; t = 1.16, df = 9, *p* = 0.28). Large numbers of scales covered almost the entire surface of the scape and pedicel (Figure 2A). Grooves were present which ran parallel to one another for the full length of the scales (Figure 2C). These scales varied in shape and size, and were penetrated by a large number of pores. Two rows of scales were located on the medial and dorsal edges of each segment (Figure 2B). The margin of scales of each row overlapped. The ventral surface of antenna possessed the majority of sensilla (Figure 2B). The shape of the scales on flagellum appeared similarly, and only a few pore structures were occasionally distributed on them (Figure 2D). The surface of flagellar segment had a reticular network of small ridges (Figure 2E). The ridges reached 1.39 ± 0.21 μm over the antennal surface (Figure 2F). Occasionally, there were some bumps of varying sizes in the middle of the ridges (Figure 2E).

### 3.2. Types of Sensilla

Various types of sensilla were found mainly on the lateral and ventral surfaces of the antennal flagellar segments. In this study, we identified nine different types of sensilla based on Schneider (1964) and Callahan (1975) as follows: sensilla trichodea (Str), sensilla chaetica (Sch), sensilla basiconica (Sba), sensilla auricillica (Sau), sensilla coeloconica (Sco), sensilla styloconica (Sst), Böhm sensilla (Bs), uniporous peg sensilla (Ups) and Sensilla squamiformia (Ssq) [33,34]. The difference of sensillum type appeared in a specific morphology, number and distribution pattern.

### 3.3. Sensilla Trichodea (Str)

**Figure 3:**
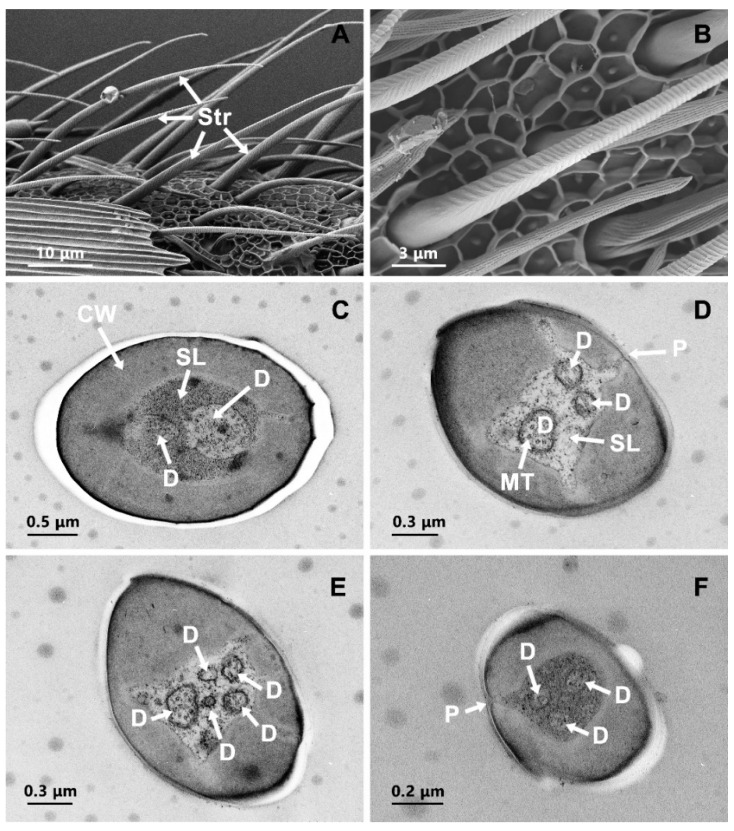
Scanning electron micrographs and transmission electron micrographs of sensilla trichodea (Str). (**A**) Distribution pattern of Str; (**B**) the surface structure of Str; (**C**) transverse section through the basal section of Str; (**D**,**E**) transverse section through the middle part of Str; (**F**) transverse section through the distal part of Str. CW: cuticle wall; D: dendrite; MT: microtubule; SL: sensilla lymph; P: pore.

Str was the most abundant type of sensillum, being present on the ventral aspect of flagellar segments in both males and females. Str was long (length 41.28 ± 5.16 μm in females and 43.54 ± 3.55 μm in males) and characterized as slightly curved, starting from the middle section toward the tip and forming an angle of 30°–45° between the antennal surface and the base of the sensilla (Figure 3A). These sensilla were unsocketed at the base. The shallow oblique grooves on the basal surface gradually transformed into transverse grooves toward the tip (Figure 3B). No difference between sexes in terms of appearance and number was found (Table 1 and Table 2). 

Analyses of cross sections revealed that two dendrites were observed in the basal section (Figure 3C). The dendrites branched into three or more dendrites in the middle region of the cuticular shaft (Figure 3C,D) and reach into the distal part of the Str (Figure 3F). The cuticular shaft of Str (a thickness of 0.42 ± 0.15 μm) were penetrated by pores, which were continuous with the sensillum lymph internally, while the shaft lacked pores in the basal section.

### 3.4. Sensilla Chaeatica (Sch)

**Figure 4:**
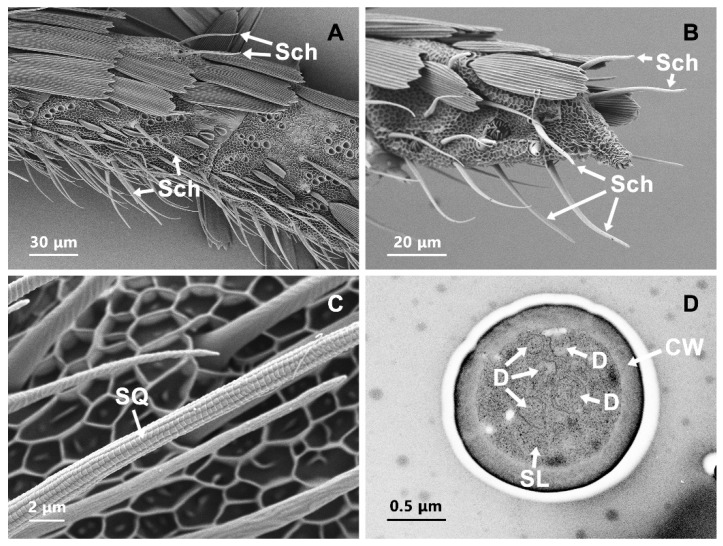
Scanning electron micrographs and transmission electron micrographs of sensilla chaetica (Sch). (**A**) Distribution pattern of Sch on flagellar segment. (**B**) distribution pattern of Sch on last flagellar segment. (**C**) the surface structure of Sch. (**D**) transverse section of Sch. CW: cuticle wall; D: dendrite; SL: sensilla lymph; SQ: squama.

Sch was long and similar to Str, and these hairs had a blunt tip and a characteristic socket at the base (Figure 4A). These sensilla formed an angle of a 60° incline toward the antennal surface and had a slightly curved rod-like morphology. Higher magnification revealed an orderly pattern of squama in the thick walls of this type of sensillum (Figure 4C). They have a terminal pore, although it is hard to observe in the SEM micrograph. They were primarily located across each flagellar section on the dorsal sides, lateral sides and ventral surfaces. There were two Sch at the middle part of the ventral surface of each segment and two Sch at the dorsal surface of each antennal segment between the scales. In addition, there were 1–2 Sch on the lateral sides of each flagellar segment (Figure 4A). Moreover, the last antennal flagellar segment had a higher number of Sch than the other flagellar segments (Figure 4B). The length of the Sch was found to be significantly longer in females than in males (Table 1). No difference in abundance between the sexes was found (Table 2). Viewed in cross-section, the cuticular wall of Sch was thick and had no pores. The distal dendrites, which contain multiple microtubules, were detected in the lymph cavity (Figure 4D).

### 3.5. Sensilla Basiconica (Sba)

**Figure 5:**
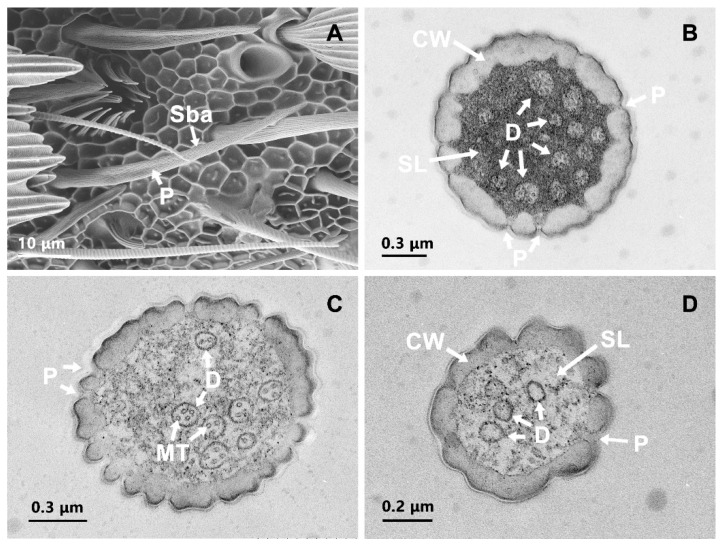
Scanning electron micrographs and transmission electron micrographs of sensilla basiconica (Sba). (**A**) The surface structure of Sba. (**B**) transverse section through the basal section of Sba. (**C**) transverse section through the middle part of Sba. (**D**) transverse section through the distal part of Sba. CW: cuticle wall; D: dendrite; MT: microtubule; SL: sensilla lymph; P: pore.

Sba was the second most common sensilla type and found mainly scattered on ventral surfaces of the flagellum. This sensilla subtype was similar in shape to Str and characterized by penetrating pores (Figure 5A). Sba was significantly shorter than Str in length in both sexes (female: t = 7.00, df = 46, *p* < 0.001; male: t = 12.27, df = 24, *p* < 0.001). These sensilla were unsocketed and curved at the base. The middle section towards the tip was almost parallel to the antennal surface (Figure 5A). The grooves on the Sba were longitudinal. Sexual dimorphism was observed, the length of Sba in females was significantly longer (t = 2.095, df = 26, *p* = 0.046) and more abundant (t = 9.243, df = 8, *p* < 0.05) in females than in males (Table 1 and Table 2). In the TEM micrographs, the surface pattern of the Sba, which consists of longitudinally arranged ridges, was discernible. The cuticular walls were extremely thin (a thickness of 0.19 ± 0.01 μm at basal and 0.13 ± 0.01 μm at middle section) (Figure 5B,C). Viewed in cross-section, multiple slit pores were observed along the thin cuticular walls (Figure 5B–D). A total of 7–12 dendrites containing numerous microtubules were observed in the lymph cavity (Figure 5B,C).

### 3.6. Sensilla Auricillica (Sau)

**Figure 6:**
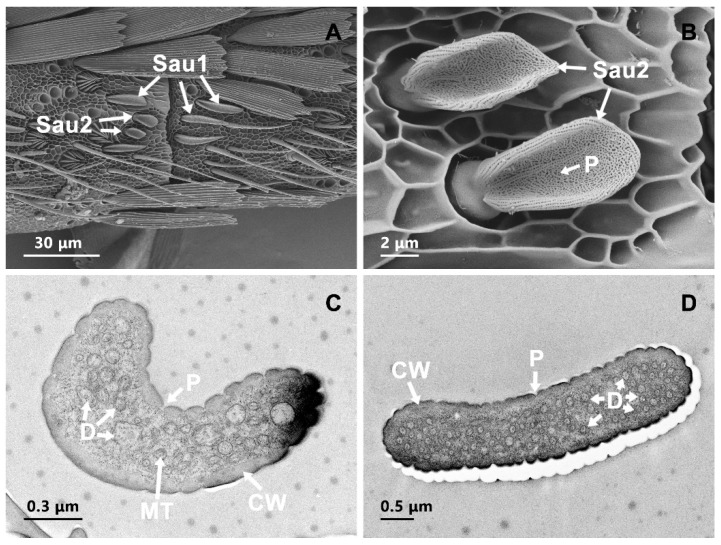
Scanning electron micrographs and transmission electron micrographs of sensilla auricillica 1 (Sau1) and sensilla auricillica 2 (Sau2). (**A**) Distribution pattern of Sau1 and Sau2; (**B**) the surface structure of Sau2; (**C**) longitudinal section of Sau2; (**D**) longitudinal section of Sau1; CW: cuticle wall; D: dendrite; MT: microtubule; P: pore.

Sau examined in this study were found near the edge of the lateral section of each flagellar segment (Figure 6A). According to the surface substructure and length, Sau can be subdivided into two subtypes: Sau1 and Sau2. Sau1 was long and had the appearance of a new leaf of Gramineae. Sau2 was short and had the appearance of a cat ear (Figure 6A). Length measurements did not show any significant difference between sexes (Table 1). Commonly, 3 to 5 Sau were grouped at the proximal and distal margins of each segment, respectively (Figure 6A). Sau1 was mainly distributed at the proximal ends, while Sau2 was distributed at the distal end. They were both multiporous sensilla (Figure 6B). According to statistical analysis, the abundance of these sensilla was significantly higher in females than that in males (Sau1: t = 4.15, df = 8, *p* = 0.003; Sau2: t = 2.553, df = 8, *p* = 0.034) (Table 2). Transverse sections showed that the cuticular wall was thin with a thickness of 0.19 ± 0.03 μm and penetrated by a dense abundance of pores, with numerous distal dendritic branches in the lymph (Figure 6C,D). 

### 3.7. Sensilla Coeloconica (Sco)

**Figure 7:**
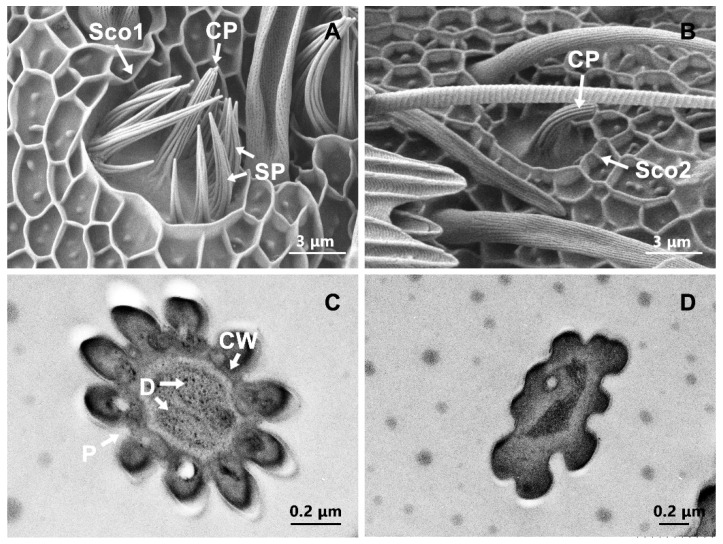
Scanning electron micrographs and transmission electron micrographs of sensilla coeloconica 1 (Sco1) and sensilla coeloconica 2 (Sco2). (**A**) Structure characters of Sco1; (**B**) structure characters of Sco2; (**C**) transverse section of the central peg; (**D**) transverse section of the spine. CP: central peg; SP: spine; CW: cuticle wall; D: dendrite; P: pore.

Two subtypes of Sco were identified: Sco1 and Sco2. Sco1 had a flower-like shape. There was a slightly curved peg stand on the shallow depression of the floor, and the central peg was surrounded by 14–16 inwards-facing spines (Figure 7A). The grooves on the cuticular surface of Sco1 were longitudinal. Sco1 was present in males and females on the ventral side and lateral side of the flagellum. There were approximately 2–5 Sco1 present in each flagellar segment (Figure 7A). The differences between the sexes for peg length of Sco1 were significant (t = 2.943, df = 8, *p* = 0.019). Similar to Sco1, Sco2 had a peg in the center without spines (Figure 7B). This sensilla subtype was rare and was found only occasionally on individual flagellar segments of males. The transmission electron micrographs revealed that Sco1 was double-walled sensillum with a thick cuticular wall and many pores, and two or three dendrites were observed in the lumen of the sensillum (Figure 7C). No dendrites were found in the spines (Figure 7D).

### 3.8. Sensilla Squamiformia (Ssq)

**Figure 8:**
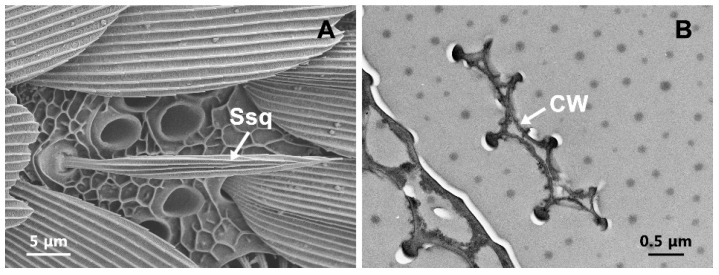
Scanning electron micrographs and transmission electron micrographs of sensilla squamiformia (Ssq). (**A**) Structure characters of Ssq. (**B**) transverse section of ssq. CW: cuticle wall.

The surface of Ssq looked like the normal scales but were smaller and narrower in size. They were inserted into a socket (Figure 8A). These sensilla were found on the scape segments and lateral sides of the flagellar segment both in male and female antennae of *T. rufivena* (Figure 2B). In most of the antennae examined, the scales were not totally removed and thus did not allow the examination of the full length of the antenna. Cross sections, as viewed by TEM, showed that the cuticle of Ssq had longitudinally arranged furrows, but wall pores were absent (Figure 8B).

### 3.9. Sensilla Styloconica (Sst)

**Figure 9:**
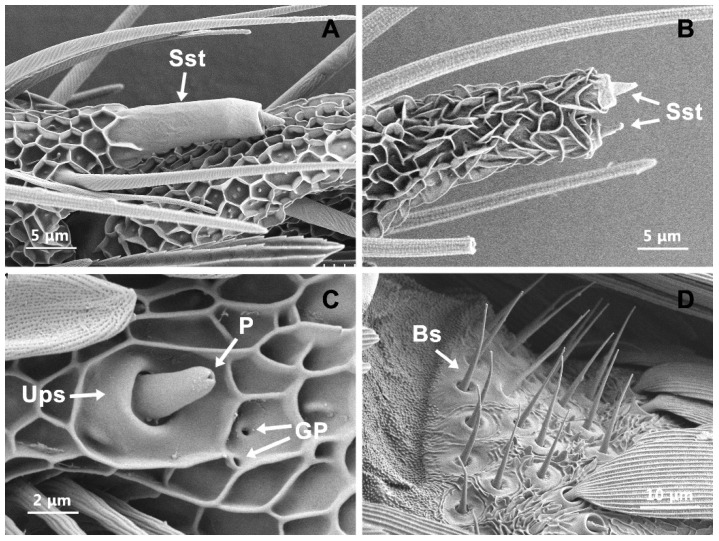
Scanning electron micrographs of sensilla styloconica (Sst), uniporous peg sensilla (Ups) and Böhm sensilla (Bs). (**A**) The surface structure of Sst on flagellar segment; (**B**) the surface structure of Sst on last flagellar segment; (**C**) the surface structure of Ups; (**D**) the surface structure of Bs. GP: glandular pore; P: pore.

Sst was thumb-like with a cone-shaped structure at the apex of the style (Figure 9A). They projected from the cuticular wall, inclining towards the apex of the segment. There was one Sst with a smooth wall at the distal margin of each flagellar segment, except for the terminal antennal segment. Two Sst with a reticular network cuticle were observed at the terminal segment (Figure 9B). These sensilla were identified in both male and female flagella. No significant differences were found between the sexes (t = 0.04, df = 5, *p* = 0.97) (Table 1 and Table 2).

### 3.10. Uniporous Peg Sensilla (Ups)

Ups comprises a round mortar-like basal socket and a small peg with a pore at the terminal tip (Figure 9C). Two glandular pores were distributed near the disc-shaped socket. This infrequent sensilla subtype was only found occasionally on individual flagellar segments of females. Because of the low abundance, it was not possible to measure the mean length, and the internal structure of this type was also not observed.

### 3.11. Böhm Sensilla (Bs)

The Bs was spine-like structures with a smooth cuticular texture. These structures would be visible in *T. rufivena* when the scales are removed. These bristles closely resembled Sch and were shorter and sharper than Sch (Figure 9D). The Bs were distributed in clusters at the joints between the scape and the pedicel and between the scape and the head (Figure 1C). The length and basal width of these sensory hairs were significantly longer in females than in males (length: t = 2.238, df = 21, *p* = 0.036; basal width: t = 3.256, df = 18, *p* = 0.004) (Table 1 and Table 2).

## 4. Discussion

Antennae are considered to be the principal organs of the insect olfactory sensory systems. Various sensilla constitute important functional elements in the detection and selection of odor molecules in the environment, and the perception of chemical, mechanical and physical signals [1,2,4,5,6,33,36]. Huang et al. have observed and analyzed seven types of sensilla using SEM, including Str (type A and type B), Sau (type A and type B), Sst, Sco, Sba, Sch and Ssq, while the internal structure and function were still not clear [32]. In this study, a total of nine sensilla types were identified, and of them, Ups and Sco2 were newly identified in *T. rufivena*. The classification of Str type B and Sba reported in Huang’s results has also been revised. In addition, we performed the TEM to further confirm the sensilla types and functions. We mainly focused on the specific ultrastructural features (e.g., wall pores, etc.), as well as variations in numbers and dimensions of some sensilla observed, which provides more details to further understand differences between sexes in the olfactory behavior of *T. rufivena*.

Sexual dimorphism of antennal length is often observed in moths. Adult males usually have well-developed antennae for sexual chemical communication [37]. In this study, the gross antennal morphology was similar between adult females and males of *T. rufivena*. There was no significant difference in antennal length between them. The antennal surface was covered with numerous scales. The scales on the scape and pedicel were not only different in shape and arrangement with those on lateral and dorsal edge of flagellar segment, but also differed with respect to the number of pores. Scales have been thought to play important roles in protecting sensilla from mechanical damages. Moreover, some researchers have speculated that the scales could trap and concentrate odorous molecules to facilitate the ability of volatile detection for insects [38,39]. Sensilla with numerous pores dispersed over the cuticular walls were thought to have an olfactory function [40]. Of these nine different sensilla types identified in our study, four types contained pores in the cuticular walls, i.e., Str, Sba, Sau and Sco, suggesting that these sensilla may be involved in chemoreception [40].

Str was the most abundant and widely distributed sensilla on the antennae of *T. rufivena*. We identified one type of Str based on its appearance and internal structure, which was inconsistent with a previous study [32]. It has been demonstrated the Str sensilla in many lepidopteran species are sensitive to pheromones and were specialized for pheromone sensing [10,41,42,43,44,45,46,47]. Moreover, some studies also demonstrated that Str was related to plant volatile detection based on the functional analysis of the non-pheromone odorant receptors. TEM observations showed the dendritic branches and the pores on the cuticle, which supported a putative olfactory function, and there was no significant difference between the sexes in Str appearance and abundance.

Sba had a similar shape to Str but with a significantly shorter size and different texture. The morphology of Sba matched the description of Str type B in the results of Huang [32]. We predicted that Sba and Str type B are the same kind of sensilla. The Sba was characterized by continuous pores and several dendrites in the lymph lumen. Based on its cross-section structures, we consider this sensilla should be identified as a Sba type. The same observation was also reported in *Eutectona machaeralis* [48], *P. interpunctella* [24], *Ostrinia nubilalis* [49] and *Tuta absoluta* [5]. Numerous pores in the cuticular walls of these sensilla indicated they play a role in olfactory functions. It is commonly accepted that the Sba are the main sensillum to sensing plant volatiles, and functionally similar in moths [2,33,41,50,51,52]. Oviposition behavior of herbivorous insects can be influenced by plant chemical substances, including secondary metabolites. These cues provide the females with vital information about the subsequent development of the host’s quality [53]. Based on sexual dimorphism in number and length of sensilla, it was speculated that Sba in *T. rufivena* had the same function and was associated with the detection of volatiles from areca inflorescence. The adult females usually lay eggs inside the gap at the base of the inflorescence before it unfolds [31]. The larger size and presence of Sba on female antennae could explain how females detect host plant odor and find suitable oviposition sites.

The Sau sensilla, which was primarily characterized by thin cuticular walls with numerous pores, were identified as the results of Huang [32]. The descriptions of this sensilla were similar to those reported for *Scoliopteryx libatrix* [54], *Loxostege sticticalis* [55] and *Cnaphalocrocis medinalis* [52]. Several studies in moths have considered Sau as olfactory receptors to plant volatiles [56,57]. Considering the fact that both Sau1 and Sau2 are more abundant in females than in males in *T. rufivena*, we assumed that they also play a vital role in the accurate positioning of areca inflorescence. Besides, some studies proposed that Sau could be responded to potential minor sex pheromone components [43,54,57]. For instance, olfactory receptor neurons located in Sau were innervated to potential minor sex pheromone components in *Cydia pomonella* [43]. The function of these sensilla need to be clarified through further investigations, such as electrophysiological and behavioral experiments.

In addition to Str, Sba and Sau, Sco was another sensilla with pores. Two types of Sco were identified on the antenna of *T. rufivena*. Sco1 was present in both sexes, and had spines outside the peg that might shield them from environmental damage [58]. Sco2, which not reported in previous results, was occasionally found on individual flagellar segments of males. These two subtypes have also been reported in *Hypsipyla grandella* [34], *Homoeosoma nebulella* [59], *O. nubilalis* [49], *Ostrinia furnacalis* [60], *Zamagiria dixolophella* [61], *L. sticticalis* [55], *C. medinalis* [52] and *P. interpunctella* [24]. It has been reported that these types of sensilla could detect not only temperature, humidity, CO_2_ and heat [59,62,63,64], but also plant volatiles. For example, some short-chain aliphatic aldehydes and acids could stimulate the neurons of these sensilla, while some monoterpene alcohols could inhibit it in *Bombyx mori* [65]. Small pores located in the longitudinal grooves and dendrites observed in the lumen indicated Sco sensilla had olfactory function in *T. rufivena*. Whether sex dimorphism in the peg length of Sco1 between sexes have an impact on olfactory function remains unknown.

The distribution and morphology of the identified Sch were similar to those on some reported Pyralidae species, including *G. mellonella* [23], *Z. dixolophella* [61], *H. nebulella* [59] and *Ephestia kuehniella* [66]. As the longest and sturdiest sensilla compared with other sensilla, Sch might have a protective role for other sensilla [67]. Because of its flexible base and terminal aperture, certain studies have demonstrated that this sensillum also serves as a contact/chemoreceptor [33,49,68]. Our findings revealed several dendrites distributed in the lymph cavity, while the thick cuticle wall was devoid of pores, which suggested a similar chemoreceptor or contact function could be attributed to this sensillum in *T. rufivena*.

Both the male and female antennae of *T. rufivena* were found to have Ssq, which are commonly present in lepidopteran insects [69]. The external morphology was also similar to the other Pyralidae, such as *H. nebulella* [59], *Z. dixolophella* [61], *P. interpunctella* [24] and *G. mellonella* [23]. These sensilla demonstrated a similar location as compared with *P. interpunctella* and *G. mellonella,* except for those located in the pedicel segments [23,24]. They could play a non-olfactory role attributed to the lack of wall pores. We predict that these sensilla may serve as mechanoreceptors, for instance, wind velocity detectors [70,71]. 

Except for the terminal antennal segment, Sst was identified and characterized singly at the distal border in both male and female antennae. Several insects are known to have these sensilla, including *O. nubilalis* [49], *Z. dixolophella* [61], *C. medinalis* [52], *P. interpunctella* [24] and *G. mellonella* [23]. This sensilla may have humidity- and temperature-sensitive functions [59,72]. In addition, the apical pore located on Sst in some lepidopteran species indicates these sensilla could have a contact chemoreceptive/gustatory function [18,38,59]. Therefore, experiments using the single-cell recording technique are necessary to determine the function of Sst, which remains unclear in this study.

The Bs, which was identified as a Sba type in the study of Huang, was specifically distributed between scape and pedicel [32]. This kind of sensilla showed a similar morphology to the description of *O. nubilalis* [49], *O. furnacalis* [60] and *Z. dixolophella* [61]. It was discovered that Bs exhibits sexual dimorphism, with females having significantly longer lengths and wider basal widths than males. Bs was proposed to have a mechanoreceptor function [33]; they sense antennal positioning and mediate movements in moths during flights [73]. In addition, studies on *C. medinalis* revealed that these sensillum might be lacking in wall pores, suggesting a non-olfactory role [52]. Whether these sensilla have the same function needs further study with the aid of single sensillum recordings.

In the female antenna of *T. rufivena*, an uniporous peg sensilla type was firstly identified. This sensilla type was described for the first time in the noctuid *Noctua pronuba* [74] and they were present in most subfamilies of Noctuidae, where they were associated with sensilla auricillica. Therefore, it is the first time that they are mentioned in Pyralidae. Some researchers predicted that these sensilla may serve as a hygroreceptor [56,75]. Further electrophysiological studies are needed to reveal its specific function.

## 5. Conclusions

From this study, we studied the ultrastructure characteristics, number and distribution of different sensilla types in both sexes to document differences between females and males. These results provide direct morphological evidence that the antennae of T. rufivena possess structures that can play a role in locating mates and host plants. This knowledge will help us to confirm the functions of the different sensilla and identify the semiochemicals mediating sexual and host-finding behavior, which are important for ongoing studies of semiochemical control for this pest.

## Figures and Tables

**Table 1:** Sizes of antennal sensilla of female and male *T. rufivena*.

Type of Sensilla	Length (µm)		Basal Width (µm)	
	Female	Male	Female	Male
Trichodea (Str)	41.28 ± 5.16	43.54 ± 3.55	2.01 ± 0.27	1.94 ± 0.19
Chaetica (Sch)	47.79 ± 5.41 *	42.62 ± 5.31	2.95 ± 0.39	2.48 ± 0.21
Basiconica (Sba)	30.99 ± 4.40 *	27.71 ± 1.53	1.56 ± 0.26	1.67 ± 0.28
Auricillica 1 (Sau1)	17.70 ± 2.04	18.79 ± 2.85	2.17 ± 0.34	2.30 ± 0.24
Auricillica 2 (Sau2)	9.16 ± 1.81	9.44 ± 1.23	2.98 ± 0.39	2.85 ± 0.23
Coeloconica 1 (Sco1)	7.45 ± 0.90 *	5.79 ± 0.67	9.02 ± 1.13	8.36 ± 1.18
Coeloconica 2 (Sco2)	-	-	-	-
Styloconica (Sst)	18.45 ± 0.27	18.43 ± 0.97	4.21 ± 0.44	4.15 ± 0.31
Böhm (Bs)	18.65 ± 2.56 *	16.20 ± 2.12	1.75 ± 0.25 *	1.48 ± 0.09
Uniporous peg (Ups)	-	-	-	-
Squamiformia (Ssq)	-	-	-	-

Note: Values are mean (±SE) value of sensilla length and basal width. * Letters indicate significant difference between sexes according to Student’s *t*-test (*p* < 0.05).

**Table 2:** Estimated number of sensilla in the antennae of male and female of *T. rufivena*.

Type of Sensilla	Mean Number	
	Female	Male
Trichodea (Str)	638.10 ± 25.97	658.43 ± 22.78
Chaetica (Sch)	235.2 ± 8.63	224.00 ± 6.57
Basiconica (Sba)	353.83 ± 6.66 *	303.83 ± 8.53
Auricillica 1 (Sau1)	178.00 ±9.34 *	156.00 ± 5.02
Auricillica 2 (Sau2)	105.60 ± 3.61 *	97.4 ± 5.31
Coeloconica 1 (Sco1)	185.60 ± 15.30	190.40 ± 8.45
Coeloconica 2 (Sco2)	NC	NC
Styloconica (Sst)	43.4 ± 1.62	43.00 ± 0.84
Böhm (Bs)	125.60 ± 8.82	121.00 ± 12.08
Uniporous peg (Ups)	NC	NC
Squamiformia (Ssq)	NC	NC

Note: Values are mean (±SE) value of sensilla number. * letters indicate significant difference between sexes according to *t*-test (*p* < 0.05). NC, not counted.

## Data Availability

The data that support the findings of this study are available from the corresponding author upon reasonable request.
